# Choroidal neovascularization secondary to ocular syphilis

**DOI:** 10.22336/rjo.2021.81

**Published:** 2021

**Authors:** Marta Paula Świerczyńska, Lech Stanisław Sedlak, Marta Anna Nowak, Dorota Wyględowska-Promieńska

**Affiliations:** * Department of Ophthalmology, Faculty of Medical Sciences in Katowice, Medical University of Silesia, Katowice, Poland; ** Department of Ophthalmology, Kornel Gibiński University Clinical Center, Medical University of Silesia, Katowice, Poland; *** Department of Histology and Cell Pathology, Faculty of Medical Sciences in Zabrze, Medical University of Silesia, Katowice, Poland

**Keywords:** inflammatory choroidal neovascularization, iCNV, neuroretinitis, ocular syphilis

## Abstract

Choroidal neovascularization (CNV) is a very rare but sight-threatening complication of ocular syphilis. We reported the case of a 51-year-old woman who presented with a 2-week history of visual loss in the right eye (RE). Fundus examination demonstrated vitritis and the optic disc margin blurring. Fundus fluorescein angiography (FFA) showed the presence of optic nerve edema, macular edema, and diffused impairment of the blood-retinal barrier with leakage areas, which led to the diagnosis of bilateral neuroretinitis. Optical coherence tomography (OCT) of the right macula evidenced irregularities of the retinal pigment epithelium (RPE), subretinal fluid and hyperreflective material. Besides, workup indicated positive serology for syphilis and the patient received combined treatment of ceftriaxone, systemic and topical steroids as well as cycloplegic medications. The woman did not consent to lumbar puncture or intravitreal anti-vascular epithelial growth factor (anti-VEGF) injection, therefore the prognosis for improvement of visual acuity is poor.

**Abbreviations:** anti-VEGF = anti-vascular epithelial growth factor, AMD = age related macular degeneration, BCVA = best corrected visual acuity, CNS = central nervous system, CNV = choroidal neovascularization, CSF = cerebrospinal fluid, FFA = fundus fluorescein angiography, FTA-ABS = fluorescent treponemal antibody absorption, HIV = human immunodeficiency virus, iCNV = inflammatory CNV, IOP = intraocular pressure, LE = left eye, MRI = magnetic resonance imaging, OCT = optical coherence tomography, OCTA = optical coherence tomography angiography, RE = right eye, RPE = retinal pigment epithelium, RPR = rapid plasma regain, TP-PA = Treponema pallidum particle agglutination, VDRL = Venereal Disease Research Laboratory

## Introduction

Syphilis is a bacterial multisystem infection caused by the spirochete Treponema pallidum. Ocular involvement occurs in about 0.6-2% of all the patients with syphilis, at any stage of disease [**1**]. The most common presentation of ocular syphilis in human immunodeficiency virus (HIV)-negative patients is posterior uveitis, whereas panuveitis is the predominant diagnosis in HIV-positive patients [**2**]. Other ocular findings described in syphilis include conjunctivitis, interstitial keratitis, episcleritis, scleritis, anterior uveitis, intermediate uveitis, retinitis, chorioretinitis, choroiditis, retinal vasculitis, papillitis, perineuritis, retrobulbar neuritis, neuroretinitis, optic atrophy, optic nerve gumma and various types of stroke syndromes [**3**-**6**]. Due to the increasing number of infections with syphilis in recent years, such manifestations may be increasingly encountered in ophthalmic practice [**1**,**4**,**7**]. 

## Case presentation

A 51-year-old Caucasian woman presented with 2-week history of visual loss and metamorphopsia in the right eye (RE). There was no previous history of eye diseases, surgery, or trauma. Her medical history included vitiligo and allergy to penicillin. In the initial examination, the best corrected visual acuity (BCVA) was 0.5/ 50 in the RE and 5/ 5 in the left eye (LE). The intraocular pressure (IOP) was 12 mmHg in the RE and 13 mmHg in the LE. Slit-lamp examination revealed pigment deposits on the lens surface after ruptured posterior synechiae in the RE. There was no evidence of a relative afferent pupillary defect. Dilated fundus examination showed the mild optic disc edema, vitreous cells (1+) in both eyes; coexisting subretinal hemorrhage with macular elevation and narrow retinal blood vessels in the RE.

**Fig. 1:**
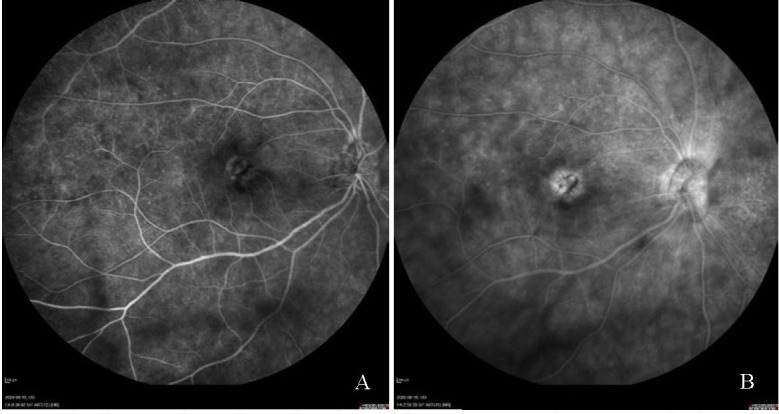
Fundus fluorescein angiography of the right eye. **A.** Hyperfluorescence in the macula in the venous phase. **B.** Petaloid pattern of leakage in the macula along with diffuse leakage of the optic disc as well as subtle leakage of the end-arterioles in the late phase

**Fig. 2:**
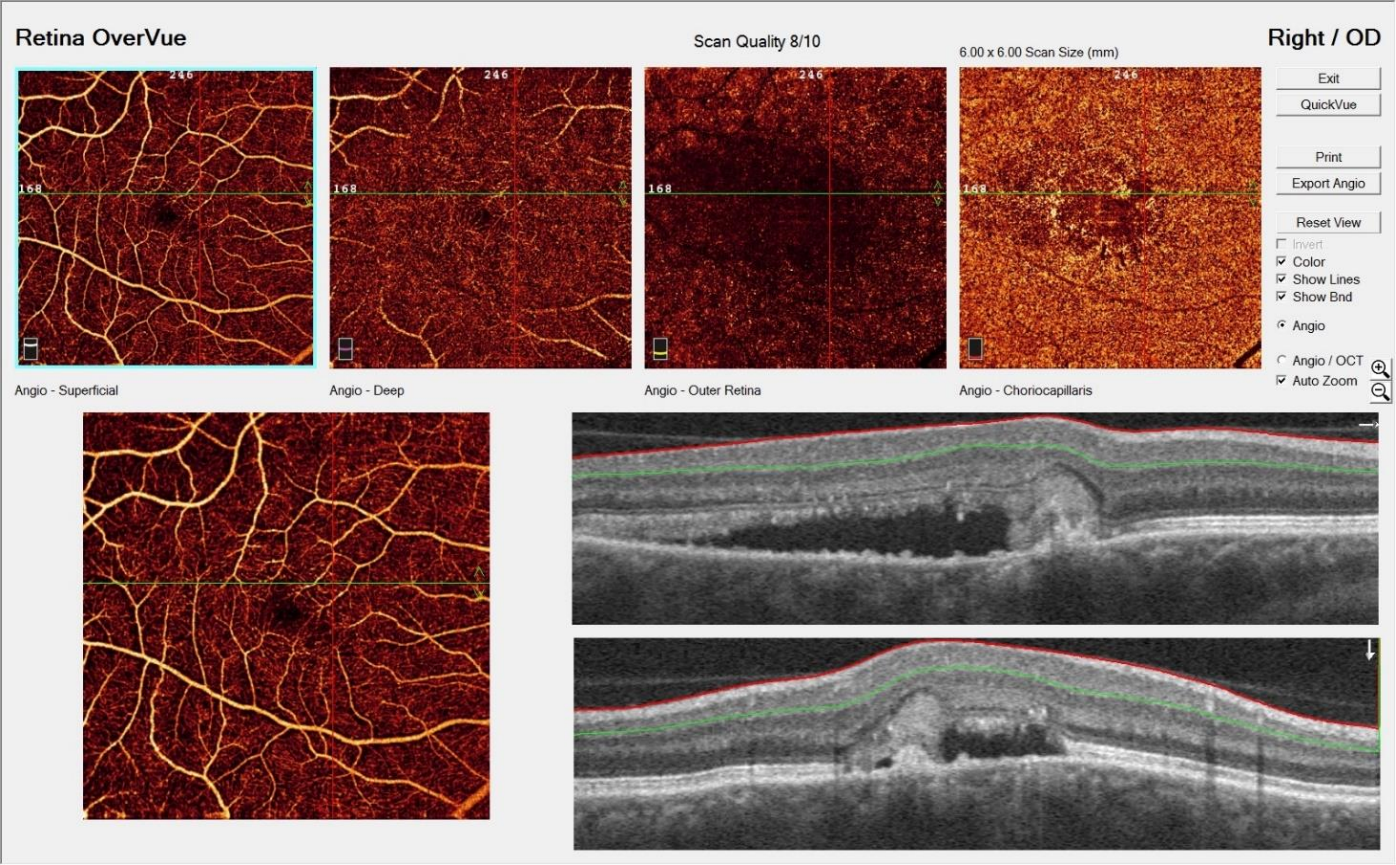
Optical coherence tomography of the right macula demonstrates the presence of a hyperreflective subfoveal lesion accompanied by accumulation of subretinal fluid

Fundus fluorescein angiography (FFA) presented blurring of the optic disc margin with contrast leakage. Hyperfluorescence increasing over time with contrast leakage indicated edema in the macula. Areas of leakage were shown in the periphery and within the posterior pole, areas of hypo-hyperfluorescence in the projection of rearrangements and defects of retinal pigment epithelium (RPE), along the vessels upstream, downstream, and temporarily. **Fig. 1 A, B** suggests bilateral neuroretinitis. Optical coherence tomography (OCT) of the right macula evidenced irregularities of the RPE, subretinal fluid and hyperreflective material, which suggested the presence of choroidal neovascularization (CNV) (**Fig. 2**). Humphrey visual field testing revealed a significant visual field defect. 

Laboratory testing results included normal complete blood test, angiotensin-converting enzyme, C-reactive protein, and erythrocyte sedimentation rate. Chest radiography showed no abnormalities. Infectious workup revealed positive IgG antibodies against cytomegalovirus and Toxoplasma gondii. What is more important, Venereal Disease Research Laboratory (VDRL) was reactive with serum titer of 1:64 and followed by positive treponemal-specific test – fluorescent treponemal antibody absorption (FTA-ABS), confirming active infection of syphilis. On further questioning the patient, she denied taking drugs, involving in high-risk sexual behaviors (she reported her last intercourse 4 years before), observing the presence of any genital ulcers or cutaneous eruptions. Testing for other sexually transmitted diseases, including hepatitis C virus and HIV, was negative. Furthermore, physical, and neurological examinations did not show any abnormalities. Lumbar puncture to obtain cerebrospinal fluid (CSF) for syphilis testing was recommended, but the patient did not consent to the examination due to its invasive nature. Magnetic resonance imaging (MRI) of the brain with contrast showed no signs of recent damage to the central nervous system (CNS). No abnormalities were found during ultrasound evaluations of the abdomen as well as carotid arteries.

Due to penicillin allergy, fourteen-day infusion of 2 g of ceftriaxone along with systemic and topical steroids, as well as cycloplegic medications were administered. Although there was a gradual decline in inflammation as treatment progressed, follow-up visual field testing showed no improvement and BCVA at discharge was 1.5/ 50 in the RE and 5/ 5 in the LE. The patient was scheduled for a check-up in the Infectious Disease Clinic to evaluate the normalization of VDRL titers. The patient was offered an intravitreal anti-vascular epithelial growth factor (anti-VEGF) injection paid out-of-pocket due to lack of insurance coverage, but the woman did not declare an interest in further therapy and was lost to follow-up.

## Discussion

Termed the great masquerader, syphilis may affect almost any ocular structure. Due to protean phenotypic manifestation, it can mimic different ocular or even systemic inflammatory diseases, leading to misdiagnoses and delays in starting effective treatment. Therefore, syphilis should be included in the differential diagnosis of ocular inflammation, especially posterior uveitis, and optic neuropathy [**3**-**6**]. Serologic diagnosis includes the nontreponemal tests (rapid plasma reagin - RPR or VDRL) however, they are nonspecific and may give false positive results due to cross-reactivity. The treponemal specific tests (Treponema pallidum particle agglutination - TP-PA or FTA-ABS) are used for confirmation [**5**-**7**]. The alternative way, preferred in high volume laboratories, is the reverse screening algorithm, when a treponemal test is performed first [**6**,**7**]. It should be emphasized that syphilis increases the risk of HIV transmission up to 5 times and co-infection is often found. Therefore, in each patient with a new diagnosis of ocular syphilis, it is advisable to test for other sexually transmitted diseases [**8**].

The Centers for Disease Control and Prevention (CDC) [**7**] recommends performing a lumbar puncture and CSF analysis for VDRL and FTA-ABS to detect neurological involvement in people with ocular syphilis. This examination is mandatory in patients with syphilitic optic neuritis and in the case of abnormal CSF results, it is repeated serially every six months to assess treatment response [**7**,**9**,**10**]. MRI, in turn, has found application in the detection of gummatous involvement of the CNS [**11**]. Patients with ocular syphilis, even with a normal CSF result, are treated according to the guidelines for neurosyphilis, with aqueous crystalline penicillin G (18 to 24 million units per day) intravenously for 10 to 14 days. In case of penicillin allergy, desensitization to penicillin in the hospital or administration of ceftriaxone intramuscularly or intravenously may be considered [**4**,**7**]. In addition, systemic steroids are often required to control ocular inflammation, but what is important, steroids should not be started before antibiotic therapy [**10**]. Moreover, it must be noted that syphilis can affect not only the nervous system and eye, but also skin, mucous membrane, bones, cardiovascular, respiratory, and digestive systems. For this reason, it is necessary to extend the evaluation of each diagnosed patient [**5**].

Inflammation is the third most common cause of CNV, right behind age related macular degeneration (AMD) and high myopia [**12**]. Most inflammatory CNV (iCNV) occur secondary to posterior uveitis or panuveitis (both infectious and non-infectious). However, the incidence differs depending on the disease entity: punctate inner choroidopathy (17-40%), idiopathic multifocal choroiditis (33%), toxoplasmosis (0.3%-19%), Vogt-Koyanagi-Harada disease (up to 9%), serpiginous choroiditis (4.7%), and presumed ocular histoplasmosis (3.8%) [**12**-**15**]. CNV is a very rare complication of posterior syphilitic uveitis, which may appear either in the active phase of the disease or only years after its onset [**3**,**16**]. Reports on syphilitic CNV are scant in literature and its natural course is still not well understood. A majority of iCNVs are type 2 (outer retinal) and it is assumed that the development of new, abnormal choroidal vessels in the subretinal space is possible due to the action of inflammation-mediated angiogenic factors and/ or secondary to degenerative disruption in the Bruch’s membrane – RPE complex [**12**,**14**,**17**]. 

Multimodal imaging plays a key role in making the correct diagnosis of suspicious lesions at the posterior pole. For a long time, FFA was the main tool to assess the presence and activity of a CNV by visualizing early hyperfluorescence in the choroidal phase and late leakage. Indocyanine green angiography is useful in visualizing feeder vessel and occult membranes. Nowadays, the fast and non-invasive OCTA examination is increasingly used [**12**,**14**,**15**]. 

The iCNV treatment methods include laser photocoagulation, photodynamic therapy (PDT) and surgical removal of the membrane. However, they have certain limitations, can cause complications, and may be associated with recurrence of CNV [**12**,**14**]. Studies have shown that most patients with iCNV respond well to intravitreal anti-VEGF injection, often in combination with local or short-term systemic steroids. In turn, long-term systemic immunosuppression is used in eyes with frequent recurrences [**12**,**15**,**17**,**18**]. In addition, it has been confirmed that people with iCNV require fewer injections than in the case of CNV secondary to other diseases, such as AMD [**12**]. Korol et al. [**13**] demonstrated that almost one-third of the participants obtained stabilization of the local state after one intravitreal injection of aflibercept and almost two-thirds managed to obtain stable vision after two injections. Bearing in mind that VEGF plays an important role as a proinflammatory mediator, anti-VEGF treatment not only inhibits angiogenesis, but also the process that is the main cause of iCNV development [**12**-**15**,**17**]. 

## Conclusion

Due to the reemergence of syphilis and its extensive range of clinical manifestations, syphilis serology should be routinely performed in each case of uveitis requiring differential diagnosis. Making an early and accurate diagnosis as well as implementation of appropriate treatment reduce the risk of permanent vision loss.

**Conflict of Interest statement**

Authors state no conflict of interest. 

**Informed Consent and Human and Animal Rights statements**

Informed consent has been obtained from all individuals included in this study. 

**Authorization for the use of human subjects**

Ethical approval: The research related to human use complies with all the relevant national regulations, institutional policies, is in accordance with the tenets of the Helsinki Declaration and has been approved by the institutional review board of the Ophthalmology Department, Kornel Gibiński University Clinical Center, Medical University of Silesia, Katowice, Poland.

**Acknowledgements**

None. 

**Sources of Funding**

None. 

**Disclosures**

None.
